# Effectiveness and safety of pembrolizumab for patients with advanced non-small cell lung cancer in real-world studies and randomized controlled trials: A systematic review and meta-analysis

**DOI:** 10.3389/fonc.2023.1044327

**Published:** 2023-02-07

**Authors:** Beibei Yang, Bing Wang, Yongbang Chen, Ning Wan, Fei Xie, Ning Yang, Liqing Lu, Weibin Xiao, Jin Yuan, Jian Li, Bo Xie, Bo Ji

**Affiliations:** ^1^ Department of Clinical Pharmacy, Baoji Central Hospital, Baoji, Shaanxi, China; ^2^ Department of Clinical Pharmacy, General Hospital of Southern Theater Command, Guangzhou, China; ^3^ School of Pharmaceutical Sciences, Southern Medical University, Guangzhou, China; ^4^ College of Pharmacy, Jinan University, Guangzhou, China; ^5^ Guangdong Branch Center, National Clinical Research Center for Geriatric Diseases (Chinese PLA General Hospital), Guangzhou, China; ^6^ Department of Oncology, General Hospital of Southern Theater Command, Guangzhou, China

**Keywords:** pembrolizumab, meta-analysis, non-small cell lung cancer, randomized controlled trials, real-world studies

## Abstract

**Background:**

Several randomized controlled trials (RCTs) have confirmed the favorable clinical benefit of pembrolizumab in advanced non-small cell lung cancer (NSCLC). However, considering the strict inclusion and exclusion criteria in clinical research, there are certain differences between patients in the real-world, it is unclear whether the findings of clinical trials are fully representative of the treatment efficacy in patients who will eventually use it. Therefore, to further comprehensively assess the efficacy and safety of pembrolizumab in NSCLC, we conducted a systematic review and meta-analysis based on the latest RCTs and real-world studies (RWSs).

**Methods:**

We systematically searched PubMed, Embase, The Cochrane Library, The Web of Science, and clinical trials.gov as of December 2021. RCTs and RWSs of patients receiving pembrolizumab monotherapy or in combination with chemotherapy for advanced NSCLC were included.

**Results:**

The meta-analysis ultimately included 11 RCTs and 26 RWSs with a total of 10,695 patients. The primary outcomes of this study were overall survival (OS), progression-free survival (PFS), objective response rate (ORR), serious adverse events (SAEs), the incidence of severe pneumonia reactions, and drug-related mortality. Direct meta-analysis results showed that in RCTs, pembrolizumab in combination with chemotherapy was superior to chemotherapy in terms of OS (HR=0.60, 95%CI:0.50-0.73), PFS (HR=0.47, 95%CI:0.38-0.58) and ORR (OR=3.22, 95%CI:2.57-4.03); pembrolizumab monotherapy was superior to chemotherapy in terms of OS (HR=0.73, 95%CI:0.66-0.80) and ORR (OR=1.90, 95%CI:1.17-3.09), but comparable to chemotherapy in terms of PFS (HR=0.83, 95%CI:0.66-1.04). The ORR values in retrospective single-arm studies were 45% (40%-51%).

**Conclusion:**

In RCTs, pembrolizumab monotherapy or in combination with chemotherapy is more effective and safer than chemotherapy for advanced NSCLC. In RWSs, ECOG PS 0-1 was shown to correlate with PFS and OS for patients with NSCLC.

## Introduction

According to the Global Cancer Statistics 2020, lung cancer remains the leading cause of cancer deaths with an incidence and mortality rate of 11.4% and 18.0%, respectively, with 2,206,771 and 1,796,144 new diagnoses and deaths, respectively (1). About 1,918,030 new cancer cases are expected to occur in the United States in 2022, with lung cancer having the third highest incidence rate in the United States, but still the highest mortality rate (2). The latest statistical report on cancer in China shows that lung cancer has the highest incidence and mortality rate (3). In addition, the 5-year survival rate for lung cancer ranges from approximately 10%~20% in most countries (4). Lung cancer includes small cell lung cancer and non-small cell lung cancer (NSCLC), of which NSCLC accounts for about 85% of all lung cancers, and most patients have locally advanced or metastatic disease at the time of diagnosis (5, 6). The traditional treatment of NSCLC is usually based on surgical resection, radiotherapy, and targeted therapy, but its therapeutic effect is poor (7). In recent years, immune checkpoint inhibitors (ICIs) targeting the PD-1/PD-L1 pathway have made breakthroughs in the treatment of NSCLC, which are more effective and safer compared with conventional treatments (8–10).

Pembrolizumab is a highly selective humanized anti-PD-1 IgG4 kappa isotype monoclonal antibody that disrupts the interaction of PD-1 with its ligand, thus showing better antitumor activity (11). In 2016, pembrolizumab was approved by the US Food and Drug Administration (FDA) for the treatment of patients with metastatic NSCLC (12). There have also been numerous clinical studies showing that pembrolizumab can provide a large clinical benefit for patients with advanced NSCLC. For example, in the KEYNOTE-024 (13) and KEYNOTE-042 (14) studies, the results of which showed improved overall survival (OS) with pembrolizumab alone in first-line untreated patients with advanced NSCLC. In addition, the results of the KEYNOTE-189 study (15) showed that pembrolizumab in combination with chemotherapy in first-line treatment of patients with metastatic NSCLC significantly prolonged OS and progression-free survival (PFS) compared with chemotherapy. In addition to the randomized controlled trials (RCTs) mentioned above, relevant studies from real-world data have also been published, and these studies likewise confirm that the actual treatment effects of pembrolizumab in a real-world setting are consistent with the comparative efficacy in RCTs (16–18).

Although a meta-analysis of pembrolizumab for advanced NSCLC has been reported currently, the sample sizes included in these studies (19–21) are small and the data from relevant RCTs have been updated over time; therefore, in this paper, the latest reported RCTs will be included, relevant data will be combined and analyzed by extraction. The results of published studies based on real-world data will be summarized on time, aiming to synthesize the available evidence on the efficacy and safety of pembrolizumab monotherapy or in combination with chemotherapy for patients with advanced NSCLC.

## Methods

### Search strategy

This meta-analysis was conducted according to the Preferred Reporting Initiative for Systematic Reviews and Meta-analyses (PRISMA) guidelines. We searched the PubMed, Cochrane Library, Web of Science, and Embase databases through December 2021 for RCTs and retrospective studies involving pembrolizumab monotherapy or in combination with chemotherapy for patients with advanced NSCLC. The keywords were “Non-small Cell Lung Cancer”, “Lung neoplasm” and “Non-small Cell Lung”. We also reviewed abstracts from the American Society of Clinical Oncology (ASCO), the World Conference on Lung Cancer (WCLC), and the European Society of Medical Oncology (ESMO). We also searched the ClinicalTrials.gov website (https://clinicaltrials.gov) to find ongoing studies and unpublished data.

### Selection criteria

Inclusion criteria: 1) Population: patients with advanced NSCLC diagnosed by histology; 2) Intervention: pembrolizumab monotherapy or in combination with chemotherapy, regardless of dose and duration; 3) Control: chemotherapy; 4) Outcome: overall survival (OS) and progression-free survival (PFS) (measured as hazard ratio(HR)), objective response rate (ORR), serious adverse events (SAEs); 5) Studies published in English. If studies were followed multiple times over time, we only report the most recent relevant data. Studies that did not meet the inclusion criteria were excluded.

### Data extraction

Data were extracted and evaluated independently by two different authors, and differences were further discussed with the third author to reach a consensus. Each clinical trial recorded the first author, year of publication, number of patients, ORR, PFS, OS, and safety outcomes, including the incidence of serious adverse events (SAEs), pneumonia (≥Grade 3), and drug-related death rate.

### The risk of bias

The risk of bias in uncontrolled studies was assessed using the non-randomized methodological item MINORS (22). RCTs were assessed using the Cochrane Risk of Bias tool (23). The quality of this study was independently assessed by two reviewers and validated by a third reviewer.

### Data analysis

Stata version 15.0 was obtained separately for ORR, 6-month progression-free survival (6m PFSr), 6-month overall survival (6m OSr), 1-year progression-free survival (1y PFSr), 1-year overall survival (1y OSr), 36-month overall survival (36m OSr), serious advanced events (SAEs), pneumonia (≥Grade 3), and drug-related mortality of the pooled data. In addition, ORR was further stratified according to tumor PD-L1 expression status, histology type and so on. Heterogeneity of the extracted data was assessed by I^2^ statistic and chi-square Q test, where I^2^≥50% (I^2^ statistic) or P ≤ 0.05 (Q test) was considered as significant heterogeneity. In the case of high potential heterogeneity, a random-effects model was used to avoid underestimating the standard error of the combined data.

## Results

### Search results

**Figure 1:**
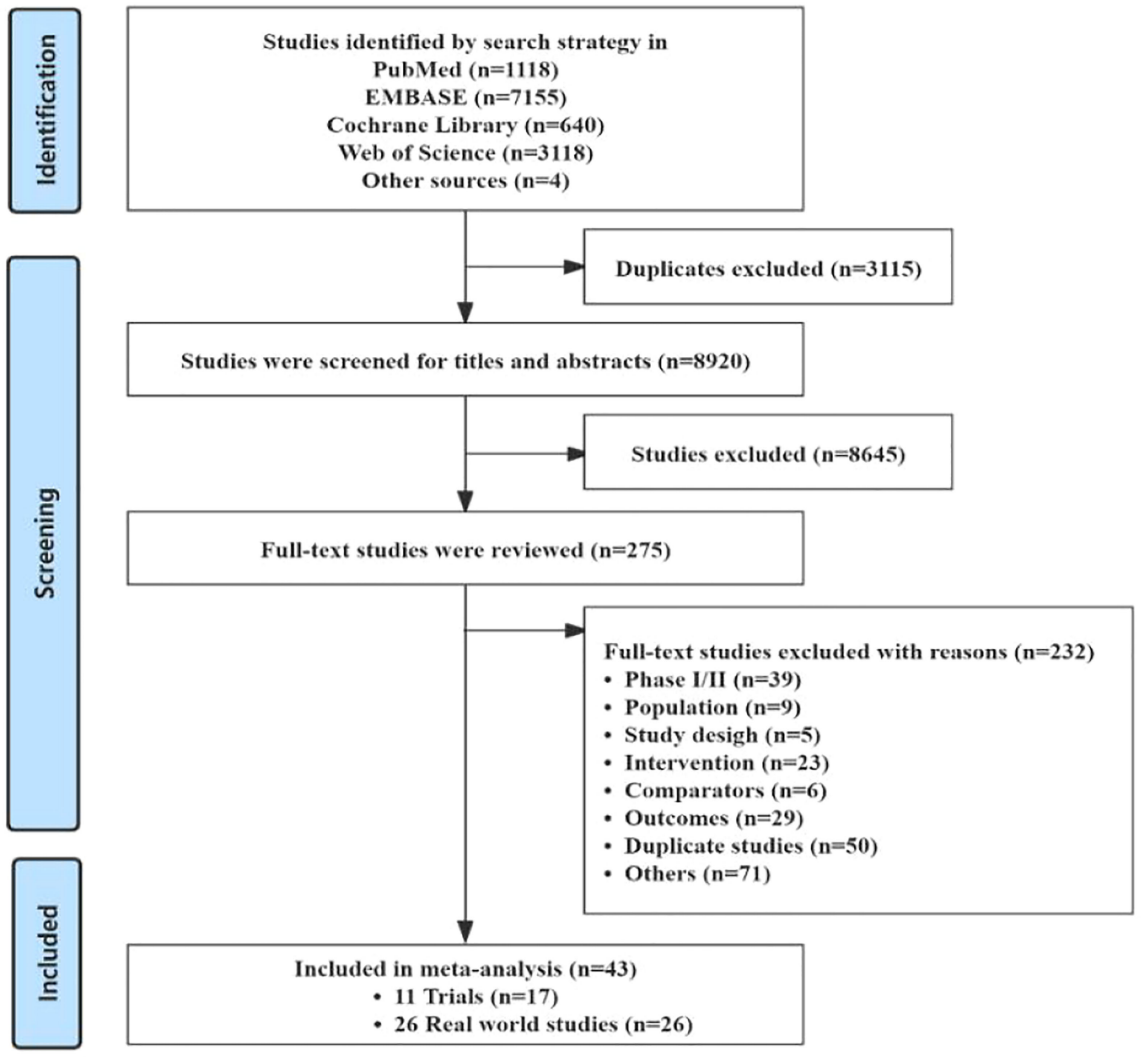
Study screening flow chart of literature selection.

A total of 12,035 articles were generated by the search strategy, of which 1,118 articles were retrieved in PubMed, 7,155 articles in EMBASE, 640 articles in the Cochrane Library, 3,118 articles in the Web of Science database, 4 articles from other sources. Finally, 11 RCTs and 26 retrospective studies were selected for combined analysis based on inclusion and exclusion criteria, respectively (The flow chart of literature screening is shown in **Figure 1**).

### Included study characteristics

Of the 11 RCTs (17 articles) included, 6 studies (7 articles) reported the efficacy and adverse effects of pembrolizumab in combination with chemotherapy for the treatment of advanced NSCLC, enrolling a total of 1,541 patients. 5 studies (10 articles) reported pembrolizumab monotherapy for the treatment of advanced NSCLC, enrolling a total of 3,299 patients. In addition, of the 26 retrospective studies included, only 3 studies were pembrolizumab in combination with chemotherapy versus chemotherapy, enrolling a total of 222 patients. The other 23 studies were pembrolizumab single-arm studies. The detailed characteristics of the included studies are shown in **Tables 1**, **2**.

**Table 1:** Characteristics of RCT studies.

References	Trial number/name	Median follow up duration (months)	No. of patients	Treatment	Histology	PD-L1 expression	ORR	OS HR (95%CI)	PFS HR (95%CI)
**Pembro + Chemo vs. Chemo**									
D. Rodríguez-Abreu (24)	NCT02578680/ KEYNOTE-189	31.0	410	Pembro+Chemo	NSq	<1% n=190;≥1% n=388;1%~49% n=186;≥50% n=202;NE n=38	All:198/410 (48.3%) <1%:42/127 (33.1%) 1%~49%:64/128 (50.0%) ≥50%:82/132 (62.1%)	0.56 (0.46-0.69)	0.49 (0.41-0.59)
			206	Placebo+Chemo			All:41/206 (19.9%) <1%:9/63 (14.3%) 1%~49%:12/58 (20.7%) ≥50%:18/70 (25.7%)		
Luis Paz-Ares (25, 26)	NCT02775435/ KEYNOTE-407	14.3	278	Pembro+Chemo	Sq	<1% n=194;≥1% n=353;1%~49% n=207;≥50% n=146;NE n=12	All:174/278 (62.6%) <1%:64/95 (67.4%) ≥1%:104/176 (59.1%)	0.71 (0.58-0.88)	0.57 (0.47-0.69)
			281	Placebo+Chemo			All:108/281 (38.4%) <1%:41/99 (41.4%) ≥1%:66/177 (37.3%)		
Mark M. Awad (27)	NCT02039674/ KEYNOTE-021	49.4	60	Pembro+PC	NSq	<1% n=44;1%~49% n=42;≥50% n=37	All:35/60 (58%) <1%:14/21 (67%) ≥1%:21/39 (54%)	0.71 (0.45-1.12)	0.54 (0.35-0.83)
			63	PC			All:21/63 (33%) <1%:4/23 (17%) ≥1%:17/40 (43%)		
Oscar Arrieta (28)	NCT02574598	8.9	40	Pembro+Doce	NR	<50% n=21;≥50% n=9	All:17/40 (42.5%)	NR	0.24 (0.13-0.46)
			38	Doce			All:6/38 (15.8%)		
Cheng Y (29)	NCT03875092/ KEYNOTE-407 in China	28.1	65	Pembro+Chemo	Sq	<1% n=48;≥1% n=72;1%~49% n=35;≥50% n=37;NE n=5	All:52/65 (80.0%)	0.44 (0.28-0.70)	0.35 (0.24-0.52)
			60	Placebo+Chemo			All:26/60 (43.3%)		
Hidehito Horinouchi (30)	NCT03950674/ KEYNOTE-189 in Japan	18.5	25	Pembro+Chemo	NSq	<1% n=20;≥1% n=16;NE n=4	All:14/25 (56%)	0.29 (0.07-1.15)	0.62 (0.27-1.42)
			15	Placebo+Chemo			All:5/15 (33%)		
**Pembro vs. Chemo**									
Martin Reck (13, 31, 32)	NCT02142738/ KEYNOTE-024	59.9	154	Pembro	Sq, NSq	≥50% n=305	≥50%:71/154 (46.1%)	0.62 (0.48-0.81)	0.50 (0.39-0.65)
			151	Chemo			≥50%:47/151 (31.1%)		
Roy S. Herbst (33–35)	NCT02220894/ KEYNOTE-010	67.4	690	Pembro	Sq, NSq	1%~49% n=591;≥50% n=442	≥1%:146/690 (21.2%) ≥50%:96/290 (33.1%)	0.70 (0.61-0.80)	0.84 (0.73-0.96)
			343	Chemo			≥1%:33/343 (9.6%) ≥50%:15/152 (9.2%)		
Tony.S.K. Mok (14, 36)	NCT02220894/ KEYNOTE-042	12.8	637	Pembro	Sq, NSq	≥1% n=1,274;≥20% n=818;≥50% n=599	≥1%:174/637 (27%) ≥20%:138/413 (33%) ≥50%:118/299 (39%)	0.82 (0.71-0.93)	1.05 (0.93-1.19)
			637	Doce			≥1%:169/637 (26.5%) ≥20%:117/405 (29%) ≥50%:96/300 (32%)		
Zhou C (37)	NCT02864394/ KEYNOTE-033	18.8	213	Pembro	NR	≥1% n=425;≥50% n=227	≥1%:44/213 (20.7%) ≥50%:32/114 (28.1%)	0.75 (0.60-0.95)	0.84(0.66-1.08)
			212	Doce			≥1%:12/212 (5.7%) ≥50%:8/113 (7.1%)		
Wu YL (38)	NCT03850444/ KEYNOTE-042 in China	33.0	128	Pembro	Sq, NSq	≥1% n=262;≥20% n=204;≥50% n=146	≥1%:40/128 (31.3%) ≥20%:34/101 (33.7%) ≥50%:29/72 (40.3%)	0.67 (0.50-0.89)	1.00(0.76-1.31)
			134	Chemo			≥1%:33/134 (24.6%) ≥20%:25/103 (25.0%) ≥50%:18/74 (24.3%)		

Pembro, Pembrolizumab; Chemo, Chemotherapy; PC, Pemetrexed plus Carboplatin; NSq, Non-Squamous; Sq, Squamous; NR, Not Reported.

**Table 2:** Characteristics of retrospective studies.

References	Year	Median follow up duration (months)	No. of patients	Treatment	Histology	PD-L1 expression	ORR	median OS month	median PFS month
Muhammad Zubair Afzal (39)	2018	NR	17	Pembro+PC	NSq	<1% n=4;1%~50% n=5;>50% n=2	8/15 (53.3%)	NR	Not reached
			37	Carbo/Peme		<1% n=9;1%~50% n=4;>50% n=4	15/37 (40.5%)		3.55
Liao JT (40)	2021	NR	49	Pembro+Peme+Platinum	ADC, Others	<1% n=9;1%~49% n=9;≥50% n=13;NE n=18	18/49 (36.7%)	Not reached	10.0 (95%CI:6.0-14.0)
			53	Beva+Peme+Platinum		<1% n=8;1%~49% n=1;≥50% n=3;NE n=41	23/53 (43.4%)	Not reached	9.2 (95%CI:7.1-11.2)
Zhang J (41)	2021	24.2	34	Pembro+Peme+cisplatin	NR	<1% n=1;1%~49% n=3;≥50% n=4;Unknown n=26	19/33 (57.6%)	23.1 (95%CI:16.6-32.8)	7.6 (95%CI:5.0-9.8)
			32	Beva+Peme+cisplatin		<1% n=4;1%~49% n=1;≥50% n=2;Unknown n=25	13/31 (41.9%)	24.2 (95%CI:16.2-32.2)	9.9 (95%CI:5.0-13.0)
Alessio Cortellini (16)	2020	14.6	1,026	Pembro	Sq, NSq	≥50%	400/899 (44.5%)	17.2 (95%CI:15.3-22.3;598 censored patients)	7.9 (95%CI:6.9-9.5;599 events)
Alessio Cortellini (42)	2020	14.8	1,010	Pembro	Sq, NSq	≥50%	394/805 (48.9%)	27.4 (95%CI:19.9-27.4;575 censored patients)	12.7 (95%CI:10.7-14.2)
Alex Friedlaender (43)	2020	8.6	302	Pembro	Sq, NSq	50%~89% n=244;>90%:n=58	202/302 (66.9%)	NR	NR
Angel Qin (44)	2017	9.2	24	Pembro	ADC, Sq, Others	NR	6/24 (25%)	NR	NR
Doran Ksienski (45)	2019	6.1	190	Pembro	Sq, NSq	1%~49% n=14;≥50% n=176	NR	13.4 (95%CI:9.7-25.1)	3.7 (95%CI:2.8-4.3)
EJ Aguilar (46)	2019	12.6	187	Pembro	ADC;Sq;Not otherwise specified	≥50%	83/187 (44.4%)	Not reached	6.5 (95%CI:4.5-8.5)
Francesco Facchinetti (47)	2020	18.2	153	Pembro	ADC;Sq;Other	50%~74%/75%~100% n=71/52;50%~89%/90%~100% n=100/23;≥50% n=30	32/153 (21%)	3.0 (95%CI:2.4-3.5)	2.4 (95%CI:1.6-2.5)
Giulio Metro (48)	2020	8.7	282	Pembro	ADC;Sq;Other	≥50%~75% n=108(38.3%);>75%~100% n=101(35.8%);≥50%, undefined n=73	104/204 (43.3%)	26.5 (95%CI:17.17-not reached)	8.9 (95%CI:5.9-12.0)
Hisao Imai (49)	2020	10.1	128 (elderly:47)	Pembro	ADC;Sq;Others	50%~74% n=19;75%~100% n=28	25/47 (53.1%)	Not reached (95%CI:10.3-not reached)	7.0 (95%CI:5.4-10.6)
Joao V Alessi (50)	2020	14.8	234	Pembro	ADC;Sq;NOS	50%~89% n=118;≥90% n=107	94/234 (40.2%)	19.8 (95%CI:16.2-not reached)	6.2 (95%CI:4.9-8.4)
Karim Amrane (51)	2019	8.2	108	Pembro	Sq, NSq	≥50%	62/108 (57.4%)	15.2 (95%CI:13.9-not reached)	10.1 (95%CI:8.8-11.4)
Kazushige Wakuda (52)	2020	NR	87	Pembro	ADC;Sq;Others	50%~74% n=28;75%~100% n=64	40/87 (46.0%)	NR	NR
Motohiro Tamiya (53)	2019	11.0	213	Pembro	ADC;Sq;Others	50%~74% n=97;75%~89% n=47;90%~100% n=69	109/213 (51.2%)	17.8 (95%CI:17.8-NA)	8.3 (95%CI:6.0-10.7)
Ryuya Edahiro (54)	2019	12.0	149	Pembro	Sq, NSq	50%~89% n=99;90%~100% n=50	75/149 (50.3%)	NR	NR
Tetsuya Sakai (55)	2020	24.4	52	Pembro	NSq	≥50%	35/52 (68%)	NR	NR
Yuichi Tambo (56)	2020	8.8	95	Pembro	ADC;Non-ADC;Sq;Adenosquamous carcinoma;Combined SCLC;Other	NR	38/95 (40.0%)	Not reached	6.1 (95%CI:3.64-8.56)
Ferréol Roborel de Climens (57)	2021	3.7	33	Pembro	ADC;Sq;Others	≥90% n=14;50%~89% n=15;1%~49% n=4	NR	4.3 (95%CI:0.9-not reached)	2.1 (95%CI:0.8-8.3)
Nikolaj Frost (18)	2021	26.9	153	Pembro	ADC;TTF-1 positive;Others;Large cell carcinoma;NOS;Sq	50%~59% n=52;60%~69% n=21;70%~79% n=31;80%~89% n=24;90%~100% n=25	74/153 (48.5%)	22.0 (95%CI:15.4-28.6)	8.2 (95%CI:5.1-1.4)
Rocío Jiménez Galán (58)	2021	23.0	88	Pembro	ADC;Sq;NSCLC poorly differentiated;Others	<90%;≥90%	28/88 (31.8%)	7.9 (95%CI:1.2-14.6)	3.9 (95%CI:2.3-5.6)
Kazutaka Hosoya (59)	2021	19.8	88	Pembro	Sq;NSq	≥50%	NR	Immature	18.4 (95%CI:13.6-22.1)
Hisao Imai (60)	2021	15.7	142	Pembro	ADC;Sq;Others	50%~89% n=85;90%~100% n=57	61/142 (42.9%)	17.4 (95%CI:12.4-31.3)	7.1 (95%CI:5.6-10.6)
Lova Sun (61)	2020	15.4	570	Pembro-based therapy	ADC;Sq;Others	<1% n=149;1%~49% n=130;≥50% n=197	167/570 (29.3%)	NR	NR
Vamsidhar Velcheti (17)	2021	21.5	283	Pembro plus pemtrexed-carboplatin	NSq	≥50% n=79;1%~49% n=77;<1% n=79;Unknown n=48	160/283 (56.5%)	16.5 (95%CI:13.2-20.6)	6.4 (95%CI:5.4-7.8)

Pembro, Pembrolizumab; Nivo, Nivolumab; PC, Pemetrexed plus Carboplatin; Peme, Pemetrexed; Carbo, Carboplatin; NSq, Non-Squamous; Sq, Squamous; NR, Not Reported; NE: Not evaluated; NA, Not available; Beva, Bevacizumab; ADC, Adenocarcinoma; NOS, not otherwise specified.

### Quality assessment of individual studies

The risk of bias for the 11 RCTs and 26 retrospective studies included in this study are summarized in **Supplementary Tables S1, S2**.

### Efficacy

#### ORR

**Figure 2:**
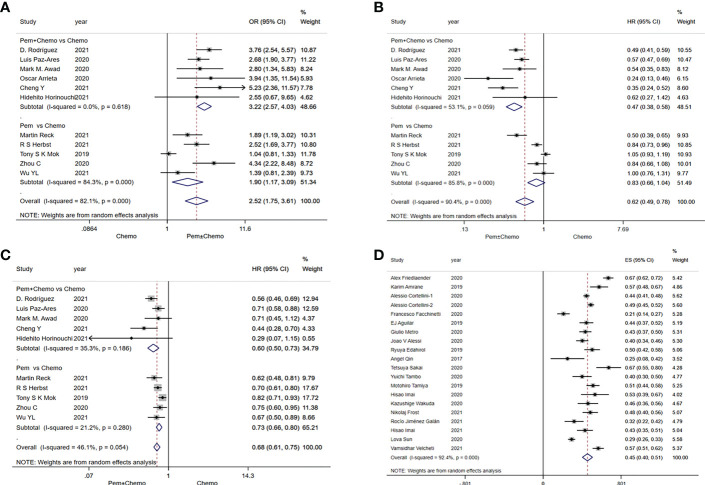
Forest plots of survival outcomes in randomized clinical trials (RCTs) and real-world studies (RWSs). **(A)** objective response rate in RCTs, **(B)** progression-free survival in RCTs, **(C)** overall survival in RCTs, **(D)** objective response rate in RWSs.

A combined analysis of relevant data extracted from RCTs showed that the ORR for treatment with pembrolizumab monotherapy or in combination with chemotherapy was significantly better than chemotherapy (OR=2.52, 95%CI:1.75-3.61) (**Figure 2A**). Pembrolizumab in combination with chemotheray was obviously better than chemotherapy in terms of ORR (OR=3.22, 95%CI:2.57-4.03). And for pembrolizumab monotherapy versus chemotherapy, the pooled ORR was OR=1.90, 95%CI:1.17-3.09 (**Figure 2A**). In addition, of the included studies of pembrolizumab monotherapy, only Martin Reck (31) had a population with PD-L1≥50%, while the remaining four studies (33, 36–38) had a population with PD-L1≥1%.

In RWSs, data were extracted from 20 included single-arm studies with a combined analysis ORR value of 45% (40%-51%) (**Figure 2D**). Analysis based on PD-L1 expression status showed a combined ORR value of 47% (42%-52%) in patients with PD-L1≥50%, 43% (36%-51%) in patients with PD-L1 50%-89%, and 53% (45%-62%) in patients with PD-L1≥90%. In addition, we also differentiated between histological types, with an ORR value of 46% (42%-51%) in non-squamous patients and 48% (43%-53%) in squamous patients (**Supplementary Figure S1**).

#### PFS and OS

In RCTs, pembrolizumab monotherapy or in combination with chemotherapy had a significant advantage over chemotherapy in terms of PFS (HR=0.62, 95%CI:0.49-0.78) (**Figure 2B**). Significant difference of PFS was observed in favor of pembrolizumab in combination with chemotherapy versus chemotherapy (HR=0.47, 95%CI:0.38-0.58). But pembrolizumab monotherapy was comparable to chemotherapy (HR=0.83, 95%CI:0.66-1.04) (**Figure 2B**). In terms of OS, pembrolizumab monotherapy or in combination with chemotherapy also has advantages over chemotherapy. Both pembrolizumab in combination with chemotherapy (HR=0.60, 95%CI:0.50-0.73) and pembrolizumab monotherapy (HR=0.73, 95%CI:0.66-0.80) significantly prolonged overall survival of patients (**Figure 2C**).

In RWSs, in univariate analysis, PS 0-1 was significantly correlated with prolongation of PFS and OS. Not using steroid (or baseline steroid<10mg) was significantly related to a prolonged PFS in univariate analysis. In multivariate analysis, PS 0-1 was identified as an independent predictor of OS prolongation. In addition, baseline steroid use was identified as an independent predictor of OS shortening in multivariate analysis (**Table 3**).

**Table 3:** Univariate analyses and multivariate analyses.

Variable	Univariate analyses		Multivariate analyses	
	Progression-Free Survival HR (95%CI)	Overall Survival HR (95%CI)	Progression-Free Survival HR (95%CI)	Overall Survival HR (95%CI)
**Performance status**				
0-1 versus 2	0.60 (0.41-0.88)	0.38 (0.29-0.51)	0.85 (0.51-1.40)	0.49 (0.28-0.87)
**Gender**				
Male versus Female	1.00 (0.90-1.11)	1.02 (0.90-1.17)	1.11 (0.95-1.30)	1.13 (0.86-1.47)
**Brain metastases**				
Yes versus No	1.24 (1.07-1.43)	1.25 (1.06-1.48)	0.98 (0.76-1.27)	1.01 (0.75-1.35)
**Age**				
<70 versus ≥70	0.96 (0.86-1.07)	0.89 (0.78-1.00)	—	—
<65 versus ≥65	1.49 (0.66-3.38)	1.23 (0.43-3.48)	1.13 (0.93-1.37)	1.13 (0.91-1.41)
**Smoking status**				
Current/Former smoker versus No-smoker	0.82 (0.48-1.38)	0.96 (0.61-1.50)	1.05 (0.67-1.65)	1.32 (1.08-1.63)
**Histology**				
Non-squamous versus Squamous	0.98 (0.87-1.10)	0.98 (0.85-1.15)	—	0.94 (0.60-1.46)
**Baseline steroids**				
No/<10mg versus ≥10mg	0.46 (0.26-0.80)	0.29 (0.06-1.29)	0.80 (0.52-1.24)	0.60 (0.40-0.90)
**IRAE**				
Yes versus No	0.76 (0.47-1.24)	0.71 (0.30-1.69)	—	—
**PD-L1 expression**				
<90% versus ≥90%	0.94 (0.69-1.29)	1.13 (0.76-1.68)	—	—
**Previous radiotherapy**				
Yes versus No	0.89 (0.48-1.65)	0.97 (0.64-1.48)	—	0.93 (0.57-1.53)

#### 6m PFSr and 12m PFSr and 24m PFSr

**Figure 3:**
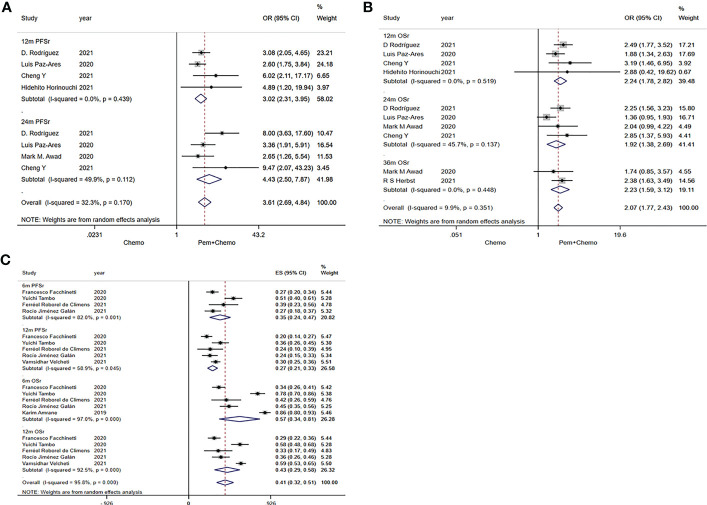
Forest plots of PFSr and OSr in randomized clinical trials (RCTs) and real-world studies (RWSs). **(A)** 12m and 24m PFSr in RCTs, **(B)** 12m, 24m, 36m OSr in RCTs, **(C)** 6m and 12m PFSr, 6m and 12m OSr in RWSs.

In RCTs, combined analysis results of 12m PFSr and 24m PFSr were 3.02 (95%CI:2.31-3.95) and 4.43 (95%CI:2.50-7.87), respectively (**Figure 3A**). Relevant data were extracted from the RWSs, and the values for the combined analyses 6m PFSr and 12m PFSr were 35% (24%-47%) and 27% (21%-33%), respectively (**Figure 3C**).

#### 12m OSr and 24m OSr and 36m OSr

The pooled values of 12m OSr, 24m OSr and 36m OSr were 2.24 (95%CI:1.78-2.82), 1.92 (95%CI:1.38-2.69), 2.23 (95%CI:1.59-3.12), respectively (**Figure 3B**). Relevant data were extracted from the four RWSs, and the combined values of 6m OSr and 12m OSr were 57% (34%-81%) and 43% (29%-58%), respectively (**Figure 3C**).

### Safety

Safety including the rate of Grade 3-5 Treatment Related Adversed Events (TRAEs), Grade 3-5 Immune Related Adversed Events (IRAEs), pneumonitis (≥Grade 3) and drug-related deaths were displayed in **Table 4**. For Grade 3-5 TRAEs and Grade 3-5 IRAEs, the incidence of pembrolizumab monotherapy and in combination with chemotherapy were 20% (15%-26%) and 64% (49%-78%) in RCTs, In the 26 retrospective studies, 2 retrospective controlled studies reported Grade 3-5 TRAEs. 3 retrospective single-arm studies mentioned grade 3-5 IRAEs. 7 retrospective studies mentioned the incidence of pneumonia (≥Grade 3), and 3 retrospective studies reported drug-related mortality. 2 retrospective controlled studies reported a Grade 3-5 TRAE rate of 51% (40%-61%) in patients with NSCLC treated with pembrolizumab in combination with chemotherapy. 3 retrospective single-arm studies reported a grade 3-5 IRAE rate of 17% (14%-21%) in patients receiving pembrolizumab monotherapy for NSCLC.

**Table 4:** Safety outcomes of the included studies.

Reference	G3–5 AEs		Pneumonitis (≥G3)		Drug-related death	
	Pembro/Pembro+Chemo	Chemo	Pembro/Pembro+Chemo	Chemo	Pembro/Pembro+Chemo	Chemo
**RCT studies**						
D. Rodríguez (24)	212/405 (52.4%)	84/202 (41.6%)	NR	NR	8/405 (2.0%)	2/202 (1.0%)
Luis Paz-Ares (26)	206/278 (74.1%)	195/280 (69.6%)	9/278 (3.2%)	3/280 (1.1%)	12/278 (4.3%)	5/280 (1.8%)
Mark M. Awad (27)	23/59 (40.0%)	19/62 (30.7%)	4/59 (6.8%)	0/62 (0%)	1/59 (1.7%)	2/62 (3.2%)
Oscar Arrieta (28)	NR	NR	0/40 (0%)	0/38 (0%)	NR	NR
Cheng Y (29)	53/65 (81.5%)	49/60 (81.7%)	0/65 (0%)	0/60 (0%)	1/65 (1.5%)	1/60 (1.7%)
Hidehito Horinouchi (30)	18/25 (72.0%)	9/15 (60.0%)	1/25 (4.0%)	2/15 (13.3%)	0/25 (0%)	0/15 (0%)
Martin Reck (31)	48/154 (31.2%)	80/150 (53.3%)	5/154 (3.3%)	1/150 (0.7%)	2/154 (1.3%)	3/150 (2.0%)
R S Herbst (33)	110/682 (16.1%)	113/309 (36.6%)	18/682 (2.6%)	2/309 (0.6%)	5/682 (0.7%)	5/309 (1.6%)
Tony S K Mok (14)	113/636 (17.8%)	252/615 (41.0%)	22/636 (3.5%)	1/615 (0.2%)	13/636 (2.0%)	14/615 (2.3%)
Wu YL (38)	25/128 (19.5%)	86/125 (68.8%)	3/128 (2.3%)	0/125 (0%)	7/128 (5.5%)	4/125 (3.2%)
**Retrospective studies**						
Liao JT (40)	25/49 (51.0%)	17/53 (32.1%)	1/49 (2.0%)	0/53 (0%)	1/49 (2.0%)	0/53 (0%)
Zhang J (41)	17/34 (50.0%)	12/32 (37.5%)	NR	NR	0/34 (0%)	0/32 (0%)
Alessio Cortellini (42)	NR	—	23/1010 (2.3%)	—	NR	—
Doran Ksienski (45)	NR	—	1/190 (0.5%)	—	NR	—
Hisao Imai (49)	NR	—	1/46 (2.1%)	—	2/47 (4.3%)	—
Motohiro Tamiya (53)	39/213 (18.3%)	—	10/213 (4.7%)	—	NR	—
Yuichi Tambo (56)	18/95 (19.0%)	—	13/95 (13.7%)	—	NR	—
Nikolaj Frost (18)	24/153 (15.7%)	—	5/153 (3.3%)	—	NR	—

Pembro, Pembrolizumab; Chemo, Chemotherapy; NR, Not Reported.

In addition, for pneumonitis (≥Grade 3), a combined analysis of 9 RCTs showed an incidence of 4% (2%-6%) in patients treated with pembrolizumab in combination with chemotherapy and 3% (2%-4%) in patients treated with pembrolizumab monotherapy, respectively. In retrospective studies, the incidence were 2% (-2%-6%) in patients treated with pembrolizumab in combination with chemotherapy. And the incidence was 3% (1%-5%) in retrospective single-arm studies.

As for drug-related death, 9 RCTs mentioned the incidence of drug-related death in patients treated with pembrolizumab, and the combined analysis incidences were 2% (1%-4%) and 2% (0%-3%) in patients treated with pembrolizumab in combination with chemotherapy and pembrolizumab monotherapy, respectively. In addition, combined data from 2 retrospective controlled studies showed that drug-related mortality occurred in patients with NSCLC treated with pembrolizumab in combination with chemotherapy at 2% (-2%-6%), while in 1 retrospective single-arm study the results showed that drug-related mortality occurred in pembrolizumab monotherapy treatment at 4% (-2%-10%). **Table 5** shows the safety results after combining the data from the RCTs and the retrospective studies, respectively.

**Table 5:** Meta-analysis of the rate of SAEs, pneumonitis, and drug-related death in combination therapy.

Studies	Items for evaluation	Rate (%)	95%CI
Pembro + Chemo in RCT studies	Grade 3-5 TRAEs	64	49-78%
	Grade 3-5 IRAEs	10	6-14%
	Pneumonitis (≥Grade 3)	4	2-6%
	Drug-related death	2	1-4%
Pembro in RCT studies	Grade 3-5 TRAEs	20	15-26%
	Grade 3-5 IRAEs	9	6-12%
	Pneumonitis (≥Grade 3)	3	2-4%
	Drug-related death	2	0-3%
Pembro + Chemo in Retrospective studies	Grade 3-5 TRAEs	51	40-61%
	Pneumonitis (≥Grade 3)	2	-2-6%
	Drug-related death	2	-2-6%
Pembro in Retrospective studies	Grade 3-5 IRAEs	17	14-21%
	Pneumonitis (≥Grade 3)	3	1-5%
	Drug-related death	4	-2-10%

### Subgroup analysis

Results of subgroup analyses are shown in **Supplementary Figures S2–S4**. In the subgroup with PD-L1<1%, pembrolizumab in combination with chemotherapy showed significant ORR, PFS, and OS advantages over chemotherapy. In the subgroup with PD-L1≥1%, pembrolizumab monotherapy or in combination with chemotherapy showed the best ORR, PFS and OS advantage over chemotherapy. In the subgroup with PD-L1 = 1-49%, pembrolizumab in combination with chemotherapy showed the best ORR, PFS and OS advantage. pembrolizumab monotherapy was superior in OS, however, pembrolizumab monotherapy was comparable to chemotherapy in PFS. In patients with PD-L1 ≥50%, pembrolizumab monotherapy or in combination with chemotherapy had a significant ORR, PFS and OS advantage over chemotherapy.

Pembrolizumab in combination with chemotherapy had a significant ORR, PFS and OS advantage over chemotherapy in both squamous and non-squamous NSCLC. Among patients receiving first-line therapy, pembrolizumab monotherapy or in combination with chemotherapy had a greater ORR, PFS and OS advantage over chemotherapy. In patients treated with second-line or more line therapy, ORR and PFS of pembrolizumab monotherapy or in combiantion with chemotherapy was higher than that of chemotherapy. In addition, among patients receiving second-line or more line regimens, pembrolizumab monotherapy was significantly better than chemotherapy in terms of OS (HR=0.71, 95%CI: 0.63-0.80).

### Sensitivity analysis

A sensitivity analysis was performed in this study to examine the effect of uncertainty on the final results. The results of the sensitivity analysis showed the included studies did not significantly affect the outcome of pembrolizumab monotherapy or in combination with chemotherapy (**Supplementary Figure S5**).

### Publication bias

Funnel plot analysis of studies of pembrolizumab monotherapy or in combination with chemotherapy treatment showed a significant asymmetric distribution (**Supplementary Figure S6A**); the Egger linear regression test verified that there was potential publication bias (**Supplementary Figure S6B**). The results of the funnel plot and the Egger linear regression test show that there was potential publication bias in our current study. The possible reason for this bias is the inclusion of two types of studies in this study, both RCTs and retrospective studies.

## Discussions

The literature included in the published meta-analysis studies on pembrolizumab for advanced NSCLC is dominated by RCTs, Zhou et al. (20) included 5 RCTs involving 1,289 patients, direct meta-analysis showed that pembrolizumab monotherapy or in combination with chemotherapy improved clinical outcomes compared with chemotherapy in patients with NSCLC. However, this study only focused on patients with first-line therapy and PD-L1≥50%. In addition, Kim et al. (21) included 4 RCTs involving 2,754 patients, their findings showed that pembrolizumab monotherapy or in combination with chemotherapy significantly improved PFS and OS in patients with advanced or metastatic NSCLC treated in the first-line. This study was published in 2019 and included a relatively small number of RCTs and patients. In addition, a small number of meta-analyses were included in RWSs. Mencoboni M et al. (62) included 32 RWSs of programmed death-1/programmed death ligand-1 (PD-1/PD-L1) for advanced NSCLC, confirming the efficacy and safety results of PD-1/PD-L1 in real-world clinical practice were similar to those in clinical trials. However, there have been no meta-analysis or systematic reviews of the efficacy and safety of pembrolizumab for the treatment of advanced NSCLC in the real world. In order to better understand the clinical efficacy of pembrolizumab in patients with NSCLC. The main innovation of our study was to analyze the efficacy of pembrolizumab treated with NSCLC in both clinical trials and real-world clinical settings. We also performed subgroup analyses for different histological types, PD-L1 expression status and different lines of treatment.

In this meta-analysis, we included 11 RCTs (n=4,840 patients) and 26 retrospective studies (n=5,819 patients). In RCTs, Pembrolizumab in combination with chemotherapy was superior to chemotherapy in ORR, PFS, and OS, but pembrolizumab monotherapy was superior to chemotherapy in ORR and OS, and no significant difference was achieved in PFS. This is consistent with the findings of Zhou et al. (20). In RCTs, we performed a combined analysis of OS, PFS, and ORR according to PD-L1 expression. In patients with PD-L1<1%, pembrolizumab in combination with chemotherapy is superior to chemotherapy in terms of ORR, PFS and OS. Pembrolizumab monotherapy or in combination with chemotherapy had a significant advantage over chemotherapy in terms of OS, PFS, and ORR in patients with PD-L1≥1%, and PD-L1≥50%. In addition, pembrolizumab in combination with chemotherapy showed the best PFS and OS benefit compared with chemotherapy in patients with PD-L1 = 1%~49%, however pembrolizumab monotherapy was comparable to chemotherapy in terms of PFS. This is similar to the results of the subgroup analysis of Passiglia et al. (63), where PD-1 in combination with chemotherapy was associated with a significant increase in ORR and PFS in patients with high PD-L1 expression, and in patients with low PD-L1 expression, PD-1 in combination with chemotherapy significantly improved OS in this group, and in the PD-L1 negative group PD-1 in combination with chemotherapy favored an increase in ORR. The difference is that our study compared pembrolizumab monotherapy or in combination with chemotherapy compared to the chemotherapy group, and Passiglia et al. (63) included PD-L1 combination chemotherapy compared to PD-L1 combined with chemotherapy compared with PD-1 alone or PD-1/PD-L1 combined with CTLA-4. In future studies, we need head-to-head studies to analyze which immunotherapy-based strategy with different PD-L1 expression is the best choice. In this study, in terms of grade 3-5 TRAEs and IRAEs, the incidence of AE was lower with pembrolizumab monotherapy compared to pembrolizumab in combination with chemotherapy in RCTs (20% vs. 64%, 9% vs. 10%). Combining the above subgroup results and AE incidence, we can recommend that in clinical practice for patients with PD-L1 ≥ 50%, pembrolizumab monotherapy seems to be an effective treatment strategy, and for PD-L1 < 50%, pembrolizumab monotherapy should be selected with caution, unless the patient reconsiders if the AE is intolerable.

In addition, the pooled ORR rate for pembrolizumab monotherapy in the 23 retrospective single-arm studies we included was 45%, which was similar to the results of the previous KEYNOTE-024 (32). It is noteworthy that the pooled ORR for patients treated with pembrolizumab (PD-L1 ≥50%) in the clinical trial of KEYNOTE-042 (14) was 39%, compared with a pooled ORR of 47% for patients with PD-L1 ≥ 50% in the retrospective study. thus showing that the ORR for patients with PD-L1 ≥50% in the retrospective study was slightly higher than in clinical trials. One possible explanation for our data is the inclusion of more patients with an ECOG PS 0 or 1 in real-world studies. We compared the safety of both RCTs and real-world studies, which showed a higher incidence of grade 3-5 TRAEs in RCTs with pembrolizumab in combination with chemotherapy than in retrospective studies (64% vs. 51%), but a significantly lower rate of grade 3-5 IRAEs with pembrolizumab monotherapy than in retrospective studies (9% vs. 17%). In addition, indirect analysis showed that the rates of pneumonia (grade ≥3) and drug-related death in the retrospective studies were comparable to those in the RCTs. A possible reason for the difference in safety results between the RCTs and the RWSs is that the number and sample size of the combined RCTs were larger than RWSs. This analysis suggests that the results of both RCTs and RWSs suggest that patients with high PD-L1 expression do benefit from first-line monotherapy with IO, seemingly confirming the results of the KEYNOTE-598 and EMPOWER-Lung 1 trials (64, 65). However, the variability in ORR and safety at the same time suggests that we cannot simply use the findings of clinical trials to assess the effectiveness and safety in real-world patients, and it is particularly important to analyze the included populations and subgroup analyses in RCTs and RWSs separately to more accurately estimate potential efficacy.

Previously, studies have confirmed that ICI monotherapy or in combination with chemotherapy are alternative options for first or second-line treatment in patients with advanced NSCLC (34, 66–68). In this study, we included 3 RCTs for patients treated in second-line or more lines. Our results showed that pembrolizumab monotherapy or in combination with chemotherapy had an advantage over chemotherapy in terms of PFS and ORR. In addition, We found that pembrolizumab monotherapy in second-line or more line therapy prolonged OS compared to chemotherapy. It is worth noting that of the 3 included RCTs of second-line or more line therapy, 1 was a phase II study of pembrolizumab in combination with chemotherapy that included patients with PD-L1≥50% and PD-L1<50%, and the clinical benefit of this study was primarily from the overall population (regardless of PD-L1 expression). Of the other 2 studies, 1 was a 5-year follow-up study of pembrolizumab monotherapy and 1 was a phase III study of pembrolizumab that included patients with PD-L1≥1% and PD-L1≥50%, with clinical benefit data from the second-line or postline study primarily for patients with PD-L1≥1%. This results also further explained the efficacy of the pembrolizumab regimen in different lines of treatment. However, in the real world, the actual clinical effect of pembrolizumab monotherapy in patients with low PD-L1 expression or posterior line therapy has not been explored in too many studies, mainly because these patients have a heavy tumor load themselves, and for these patients, the main purpose of treatment is to rapidly reduce the tumor volume, prevent excessive disease progression, and increase progression-free survival. However, in patients with low PD-L1 expression or second-line patients, immune checkpoint inhibitor monotherapy may cause rapid disease progression, so in clinical practice, this treatment option is less in patients with low PD-L1 expression.

Numerous clinical trial studies have demonstrated the effectiveness of pembrolizumab in the first-line treatment of patients with PD-L1 expression-positive NSCLC. but its clinicopathological correlates have not been concluded in RCTs. In this study, we explored the variables of interest in terms of PFS and OS. In univariate analysis,we found that PS 0-1 was confirmed to be associated with prolonged PFS and OS, whereas gender, age (<70 or ≥70 and <65 or ≥65), smoking status, histological type, immune-related adverse effects and radiotherapy were not associated with PFS and OS. In multivariate analysis, PS 0-1 and not using steroid (or baseline steroid<10mg) were confirmed as independent relevant predictors of OS prolongation, whereas gender, brain metastasis status, age (<65 or ≥65), histological type and were not confirmed as independent predictors of PFS and OS. Although the above results explain the clinically relevant factors related to OS and PFS. However, further confirmation regarding the validity of the clinical subgroups is needed in the future with more clinical trial studies.

The strength of this meta-analysis was that we included the most recent relevant data from more comprehensive RCTs and retrospective studies, directly comparing the clinical efficacy and safety of pembrolizumab monotherapy or in combination with chemotherapy versus conventional chemotherapy. Further provides evidence-based medical evidence for the superior clinical benefit of pembrolizumab in patients with NSCLC. Previously published RCTs, although reporting better survival and response rates for pembrolizumab in advanced NSCLC, may have resulted in the exclusion of some patients from the oncology group due to the strict inclusion and exclusion criteria for screening patients in RCTs. However, retrospective studies from real-world data are beneficial in providing further evidence of clinical benefit for a broader range of oncology patients, including those excluded from RCTs. Also, by comparing the combined clinical efficacy and safety results of the respective RCTs and retrospective studies, it provides a basis for more accurate estimation of potential patient outcomes in clinical practice.

There are some limitations in the present study. First, due to the lack of data related to tumor mutation burden analysis in the included RCTs and retrospective studies, the current study did not distinguish the relationship of tumor mutation burden and the efficacy of pembrolizumab monotherapy or in combination with chemotherapy treatment regimens. Second, Most of the RWSs included in this study were single-arm studies of pembrolizumab monotherapy, and there was a lack of controlled studies of pembrolizumab monotherapy or combination chemotherapy versus chemotherapy in the real world. RWSs may have heterogeneity in design or patient selection, lack of standardization in treatment regimens, or some bias in the different data collection processes, among other factors, which may have affected the results. And most of RWSs were conducted on patients with high PD-L1 expression, but not on patients with low PD-L1 expression, so more studies are needed to further validate the benefits of pembrolizumab in this population. Third, the study has not analyzed the efficacy and safety of immune monotherapy, immune combination chemotherapy, and dual immune therapy under different PD-L1 expression scenarios to determine which immune-based strategy is the best choice.

## Conclusion

In conclusion, this study further explains the superior clinical efficacy and acceptable toxicity of pembrolizumab monotherapy or in combination with chemotherapy regimens in patients with NSCLC, further providing evidence to support the use of pembrolizumab in clinical practice. In addition, in univariate analyses of RWSs, PS 0-1 appeared to be associated with PFS and OS.

## Data availability statement

The original contributions presented in the study are included in the article/**Supplementary Material**. Further inquiries can be directed to the corresponding authors.

## Author contributions

BY, BW and NW proposed the idea and designed the study. BW and YC performed the literature search and data extraction. FX, NY, LL, WX, and JY compiled and analyzed the data, and developed the figures and tables. BY and BW wrote the manuscript. JL, BX, BJ, and NW guided the study and the manuscript. All authors contributed to the article and approved the submitted version.
