# Supplementary material for: Effectiveness and safety of pembrolizumab for patients with advanced non-small cell lung cancer in real-world studies and randomized controlled trials: A systematic review and meta-analysis
Source: Front Oncol. 2023 Feb 7;13:1044327. doi: 10.3389/fonc.2023.1044327 (PMC9942927; doi:10.3389/fonc.2023.1044327)
Supplement: Supplementary file 1 [file DataSheet_1.pdf]

Supplemental Methods

Search strategies and number of studies yielded from each database

PubMed：1,118 results

(("Carcinoma, Non-Small-Cell Lung"[Mesh]) OR (((((((Carcinoma[Title/Abstract]) OR (Non Small Cell Lung[Title/Abstract])) OR (Lung Carcinoma[Title/Abstract])) OR (Non-Small-Cell[Title/Abstract])) OR (Non-Small-Cell Lung Carcinomas[Title/Abstract])) OR (Lung Neoplasms[Title/Abstract])) OR (Pulmonary Neoplasms[Title/Abstract]))) AND (("pembrolizumab" [Supplementary Concept]) OR ((((SCH-900475[Title/Abstract]) OR (Keytruda[Title/Abstract])) OR (MK-3475[Title/Abstract])) OR (lambrolizumab[Title/Abstract])))

Embase：7,155 results

('pembrolizumab'/exp OR 'sch-900475':ab,ti OR 'keytruda':ab,ti OR 'MK-3475':ab,ti OR 'lambrolizumab':ab,ti) AND ('non small cell lung cancer'/exp OR 'lung neoplasm':ab,ti OR 'non small cell lung carcinoma':ab,ti OR 'NSCLC':ab,ti OR 'lung carcinoma':ab,ti OR 'non-small-cell lung carcinomas':ab,ti OR 'pulmonary neoplasms':ab,ti)

Cochrane：640 results; 639 trials

#1 MeSH descriptor: [Carcinoma, Non-Small-Cell Lung] explode all trees

#2 "Non Small Cell Lung" OR "Non Small Cell Lung Carcinomas" OR "Non-Small-Cell" OR "Lung Neoplasms" OR "NSCLC"

#3 "pembrolizumab" OR "lambrolizumab" OR "Keytruda" OR "MK-3475"

#4 (#1 OR #2) AND #3

Web of Science：3,118 results

TS=((“Non small Cell Lung Cancer” OR “lung neoplasm” OR “Non Small Cell Lung Carcinoma” OR “NSCLC”) and (“Pembrolizumab” OR “Keytruda”))

Supplemental **TABLE S1** Bias risk evaluation of randomized controlled trials included in the meta-analysis.

Reference	Random sequence generation	Allocation concealment	Blinding of participants and personnel	Blinding of outcome assessment	Incomplete outcome data	Selective reporting	Other sources of bias
D. Rodríguez-Abreu[2021]	Low risk	Low risk	Low risk	Low risk	Low risk	Low risk	Unclear risk
Luis Paz-Ares [2018]	Low risk	Unclear risk	Low risk	Low risk	Low risk	Low risk	Unclear risk
Mark M. Awad, MD[2020]	Low risk	Low risk	Low risk	Low risk	Low risk	Low risk	Unclear risk
Oscar Arrieta, MD, MSc[2020]	Low risk	Low risk	Low risk	Unclear risk	High risk	Low risk	Unclear risk
Ying Cheng[2021]	Low risk	Low risk	Low risk	Low risk	Low risk	Low risk	Unclear risk
Hidehito Horinouchi [2021]	Low risk	Unclear risk	Low risk	Low risk	Low risk	Low risk	Unclear risk
Martin Reck [2016]	Low risk	Low risk	Low risk	Low risk	Low risk	Low risk	Unclear risk
Tony S K Mok[2019]	Low risk	Low risk	Low risk	Low risk	Low risk	Low risk	Unclear risk
R S Herbst [2021]	Low risk	Low risk	Low risk	Low risk	Low risk	Low risk	Unclear risk
Zhou C [2020]	Low risk	Unclear risk	Low risk	Low risk	Low risk	Low risk	Unclear risk
Yi-Long Wu[2021]	Low Risk	Unclear Risk	High Risk	Low Risk	Low Risk	Low risk	Unclear risk

Supplemental **TABLE S2** Bias risk evaluation of the retrospective studies included in the meta-analysis.

Reference	A clearly stated aim	Inclusion of consecutive patients	Prospective collection of data	Endpoints appropriate to the aim of the study	Unbiased assessment of the study endpoint	Follow-up period appropriate to the aim of the study	Loss to follow up less than 5%	Prospective calculation of the study size	Score
Muhammad Zubair Afzal[2018]	2	2	0	1	0	1	2	2	18
Jiatao Liao[2021]	2	2	0	2	0	1	2	2	19
Jie Zhang[2021]	2	2	0	2	0	2	2	2	19
Alex Friedlaender [2020]	2	2	0	2	0	1	2	2	11
Doran Ksienski [2019]	2	2	0	1	0	1	2	2	10
Karim Amrane [2019]	2	2	0	2	0	1	2	2	11
Alessio Cortellini [2020]-1	2	2	0	2	0	2	1	2	11
Alessio Cortellini [2020]-2	1	2	0	2	0	2	1	2	10
Francesco Facchinetti [2020]	1	2	0	2	0	2	2	2	11
EJ Aguilar [2019]	2	2	0	2	0	2	2	2	12
Giulio Metro[2020]	2	2	0	2	0	1	2	2	11
Joao V Alessi[2020]	1	2	0	2	0	2	2	2	11
Ryuya EdahiroI [2019]	2	2	0	1	0	2	2	1	10
Angel Qin[2017]	2	2	0	1	0	1	2	1	9
Tetsuya Sakai[2020]	2	2	0	2	0	2	2	2	12
Yuichi Tambo[2020]	2	2	0	2	0	1	2	2	11
Motohiro Tamiya [2019]	2	2	0	2	0	1	2	2	11
Hisao Imai[2020]	2	2	0	2	0	1	2	2	11
Kazushige Wakuda, MD[2020]	2	2	0	2	0	1	2	2	11
Ferréol Roborel de Climens [2021]	2	2	0	2	0	1	2	2	11
Nikolaj Frost-2021	2	2	0	2	0	2	2	2	12
Rocío Jiménez Galán[2021]	2	2	0	2	0	2	2	1	11
Kazutaka Hosoya [2021]	2	2	0	2	0	2	1	1	10
Hisao Imai[2021]	2	2	0	2	0	2	1	2	11
Lova Sun[2020]	2	2	0	2	0	2	2	1	11
Velcheti [2021]	2	2	0	2	0	2	2	1	11

Subgroup analysis of ORR in retrospective studies.

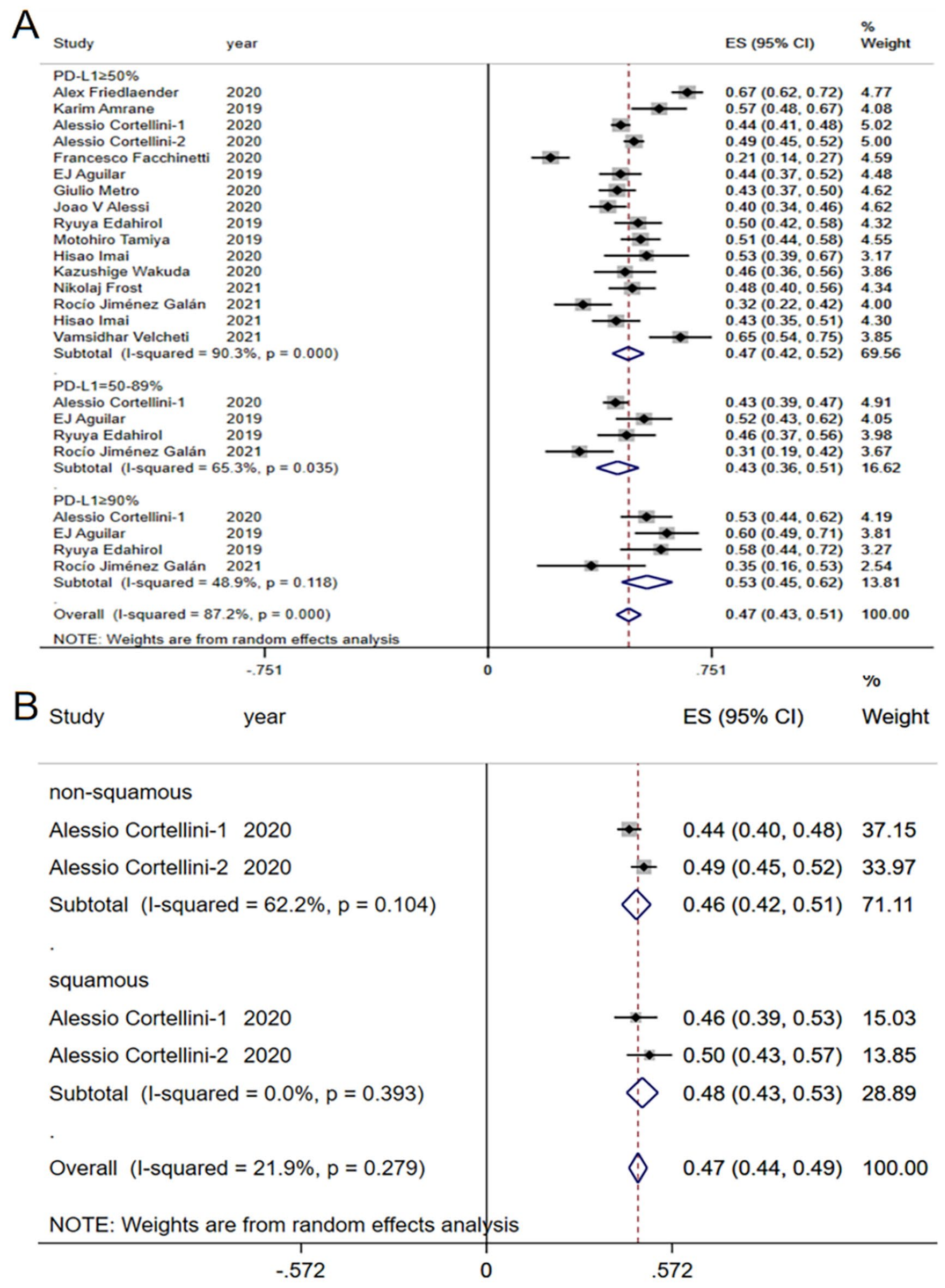

FIGURE S1

Forest plot of odds ratio(OR) of overall response rate (ORR) associated with Pembrolizumab in real-world studies (RWSs) with **A (**programmed death-ligand 1(PD-L1) subgroups), **B** (ECOG-PS subgroups), **C** (squamous or non-squamous subgroups), **D** (brain metastases or no-brain metastases subgroups), **E** (male or female subgroups).

Subgroup analysis of ORR in RCT studies

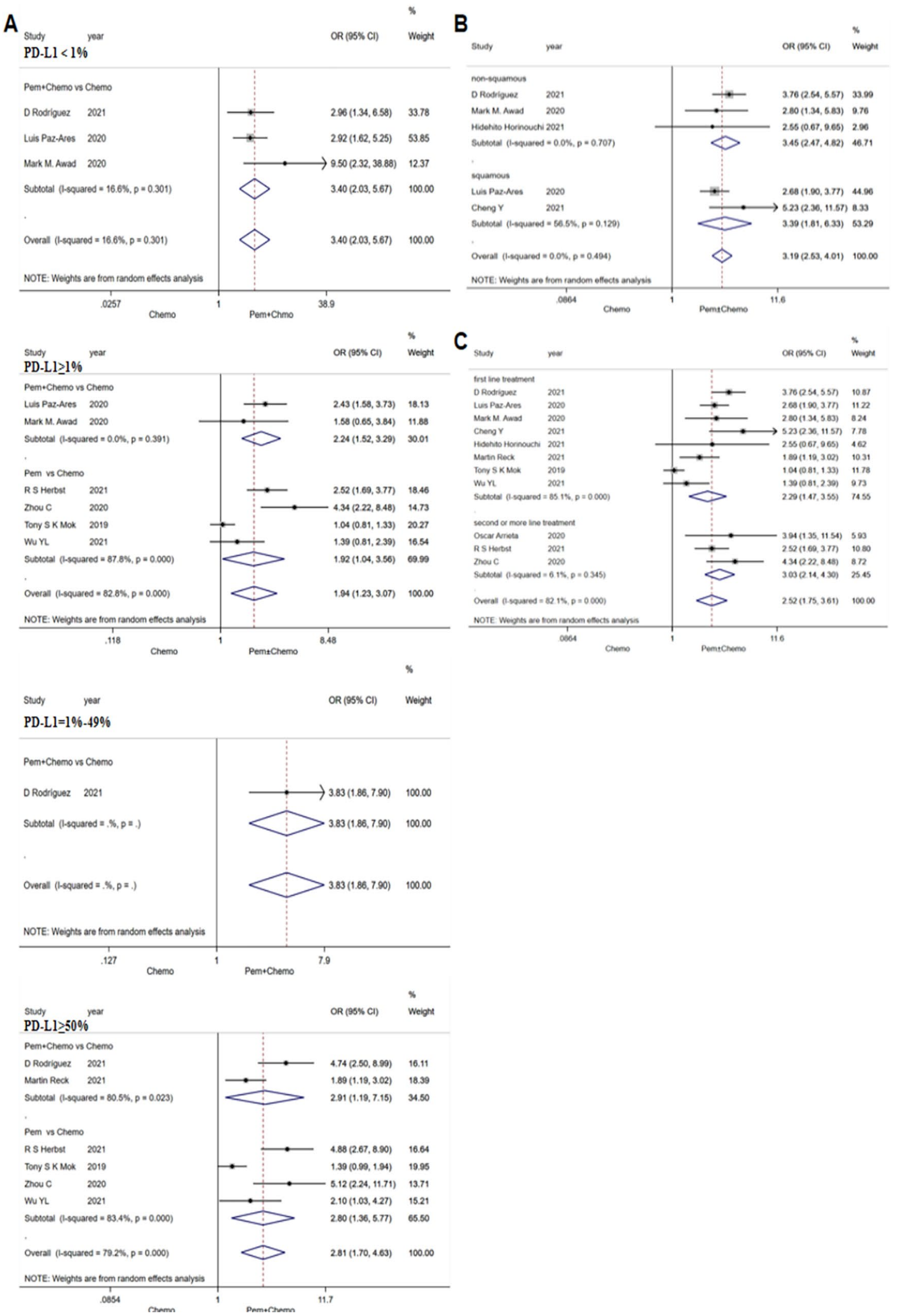

FIGURE S2

Forest plots of odds ratio(OR) of overall response rate (ORR) associated with Pem±Chemo vs Chemo in randomized clinical trials (RCTs) with **A**(programmed death-ligand 1(PD-L1) subgroups), **B**(squamous or non-squamous subgroups), **C**(first line treatment or second or more line treatment subgroups). Pem, pembrolizumab Chemo, chemotherapy.

Subgroup analysis of PFS in RCT studies

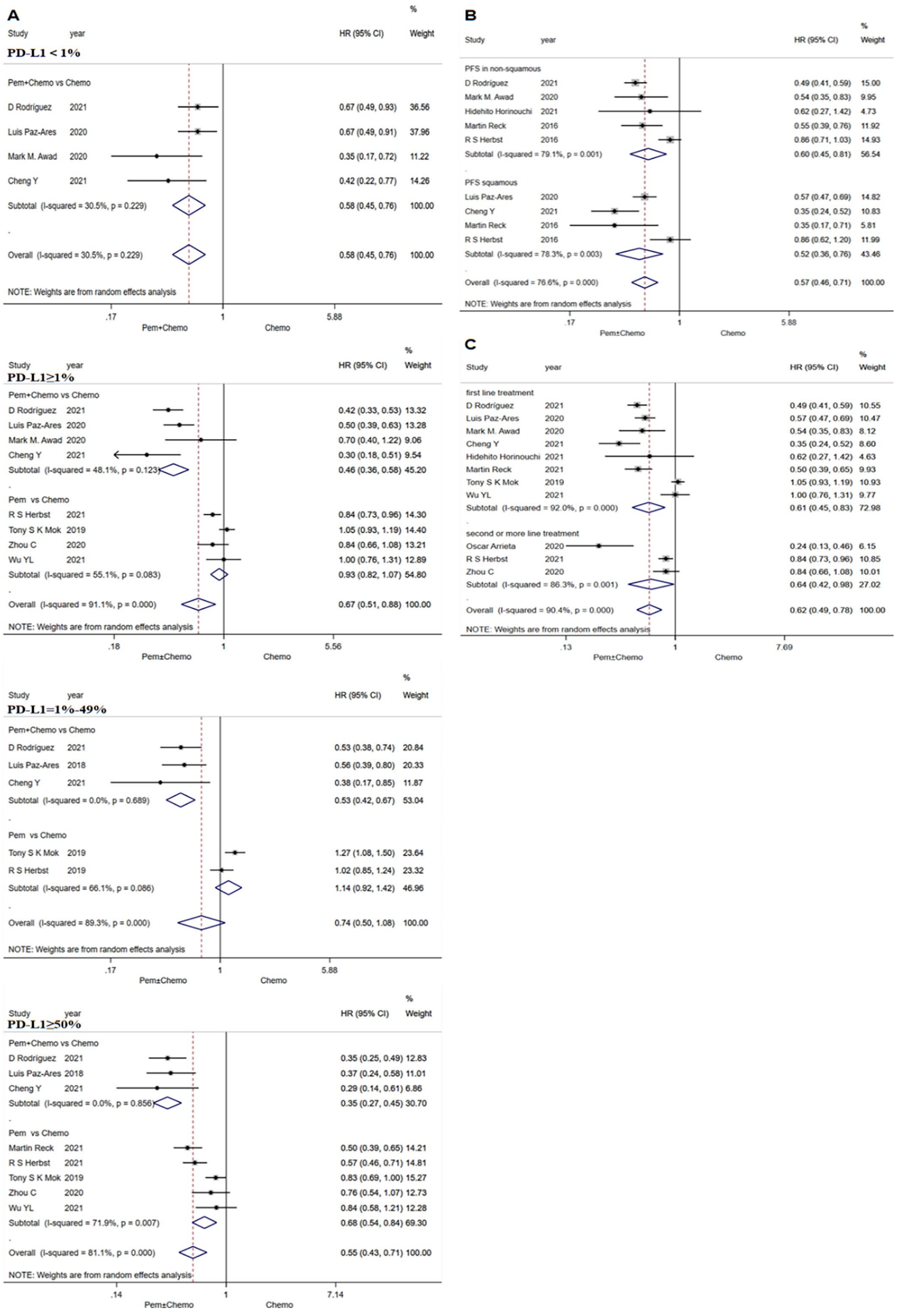

FIGURE S3

Forest plots of hazard ratio (HR) of progression-free survival (PFS) associated with Pem±Chemo vs Chemo in randomized clinical trials(RCTs) with **A** (programmed death-ligand 1(PD-L1) subgroups of PFS), **B** (squamous or non-squamous subgroups of PFS), **C** (first line treatment and second or more line treatment subgroups of PFS).

Subgroup analysis of OS in RCT studies

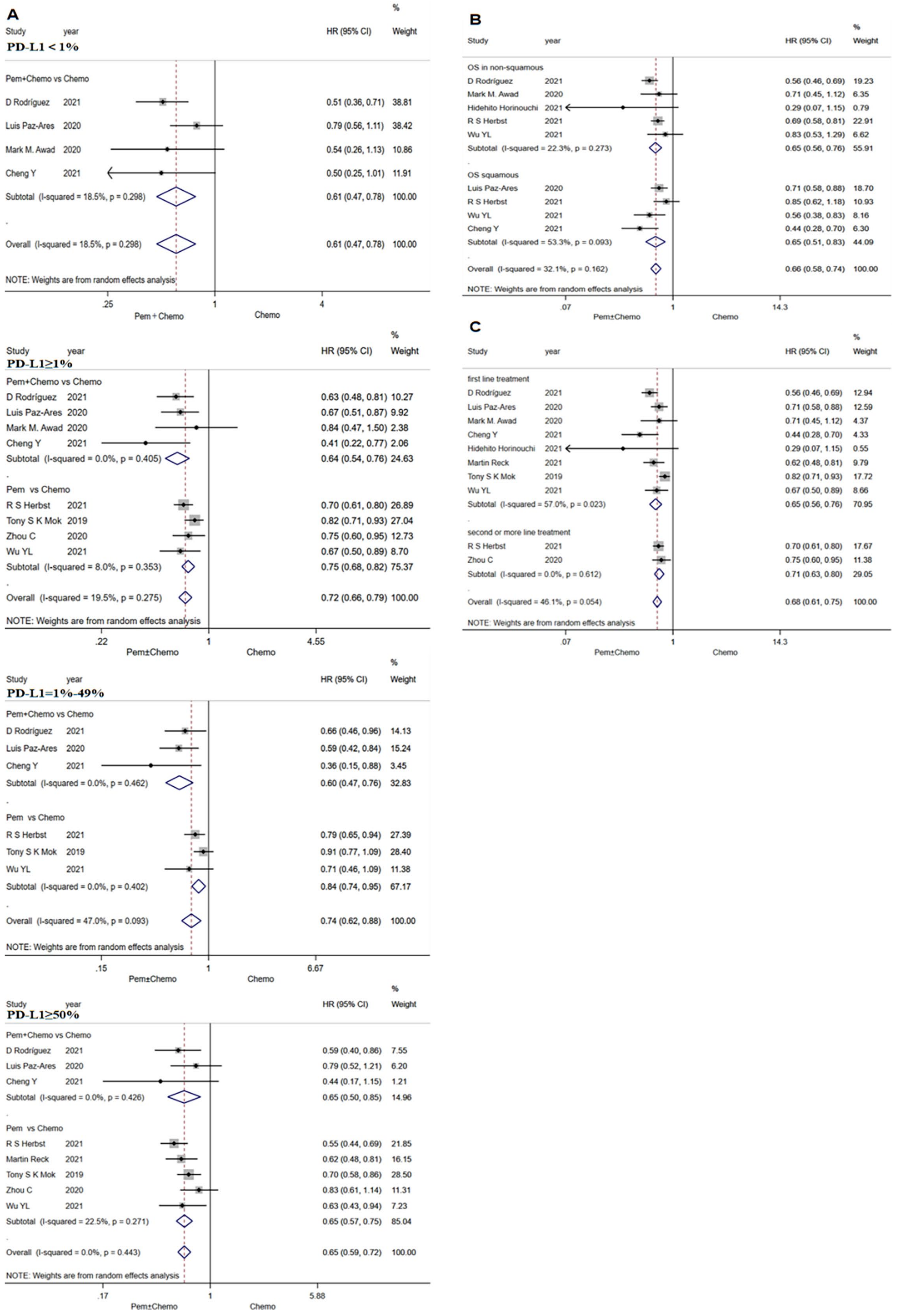

FIGURE S4

Forest plots of hazard ratio (HR) of overall survival (OS) associated with Pem±Chemo vs Chemo in randomized clinical trials(RCTs) with **A** (programmed death-ligand 1(PD-L1) subgroups of OS), **B (**squamous or non-squamous subgroups of OS), **C** (first line treatment and second or more line treatment subgroups of OS). Pem, pembrolizumab; Chemo, chemotherapy.

Sensitivity analysis of ORR for Pembrolizumab monotherapy or plus chemotherapy

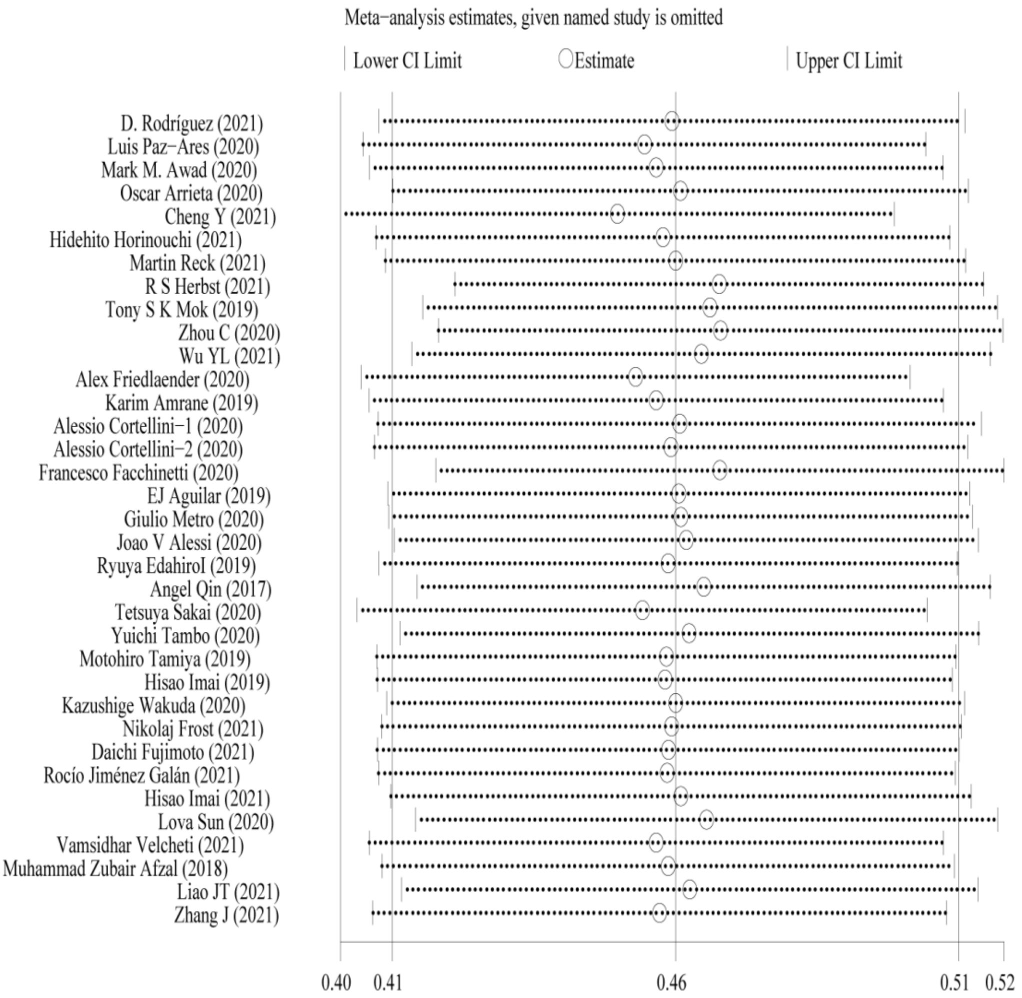

FIGURE S5

Sensitivity analysis of ORR for Pembrolizumab monotherapy or plus chemotherapy.

Begg's and Egger linear regression test of ORR for Pembrolizumab monotherapy or plus chemotherapy

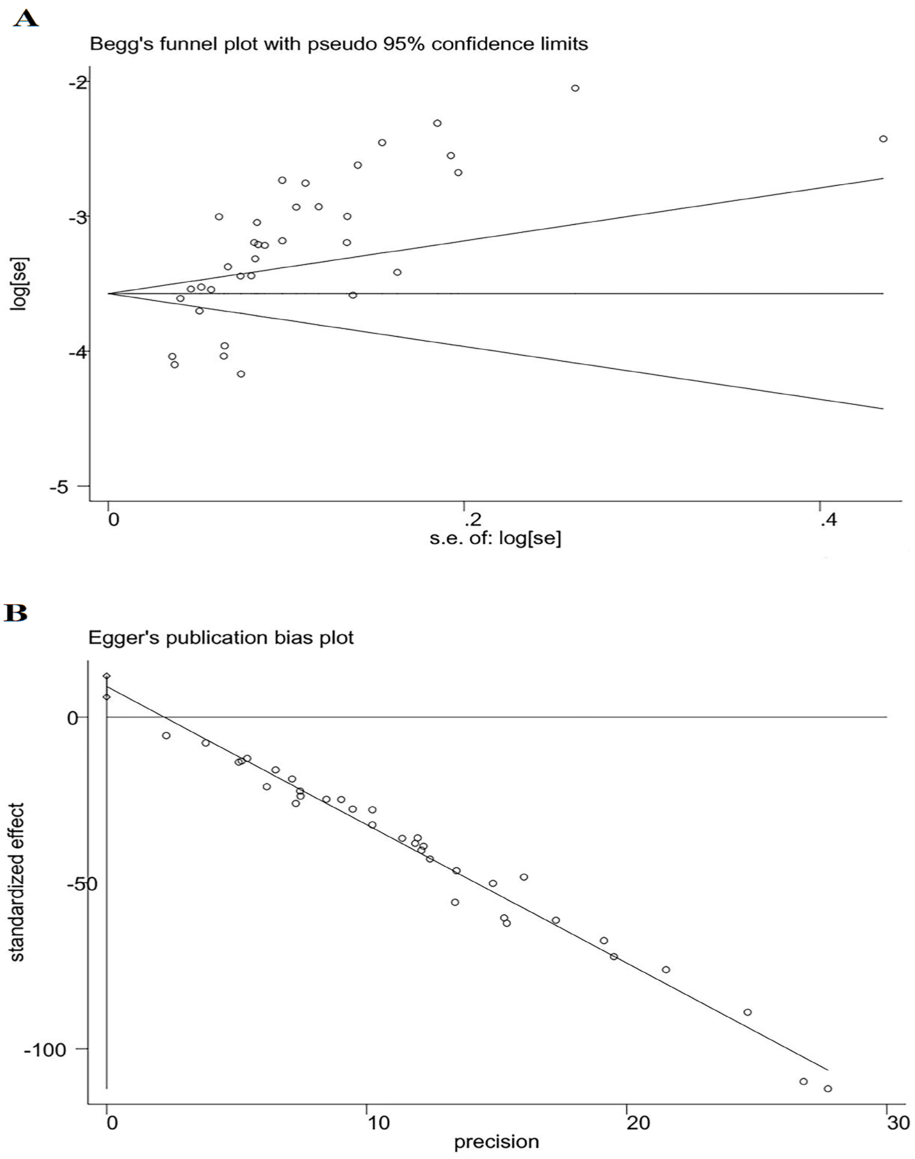

FIGURE S6

(**A**) Begg's test of ORR for Pembrolizumab monotherapy or plus chemotherapy, (**B**) Egger's linear regression test of ORR for Pembrolizumab monotherapy or plus chemotherapy.
